# P7C3-A20 treatment one year after TBI in mice repairs the blood–brain barrier, arrests chronic neurodegeneration, and restores cognition

**DOI:** 10.1073/pnas.2010430117

**Published:** 2020-10-21

**Authors:** Edwin Vázquez-Rosa, Min-Kyoo Shin, Matasha Dhar, Kalyani Chaubey, Coral J. Cintrón-Pérez, Xinmiao Tang, Xudong Liao, Emiko Miller, Yeojung Koh, Sarah Barker, Kathryn Franke, Danyel R. Crosby, Rachel Schroeder, Josie Emery, Terry C. Yin, Hisashi Fujioka, James D. Reynolds, Matthew M. Harper, Mukesh K. Jain, Andrew A. Pieper

**Affiliations:** ^a^Harrington Discovery Institute, University Hospitals Cleveland Medical Center, Cleveland, OH 44106;; ^b^Department of Psychiatry, Case Western Reserve University, Cleveland, OH 44106;; ^c^Geriatric Research Education and Clinical Centers, Louis Stokes Cleveland Veterans Affairs Medical Center, Cleveland, OH 44106;; ^d^Case Cardiovascular Research Institute, Department of Medicine, Case Western Reserve University, Cleveland, OH 44106;; ^e^Harrington Heart and Vascular Institute, University Hospitals Cleveland Medical Center, Cleveland, OH 44106;; ^f^The Department of Psychiatry, University of Iowa, Iowa City, IA 52242;; ^g^Cryo-Electron Microscopy Core, Case Western Reserve University School of Medicine, Cleveland, OH 44106;; ^h^Institute for Transformative Molecular Medicine, School of Medicine, Case Western Reserve University, Cleveland, OH 44106;; ^i^Department of Anesthesiology and Perioperative Medicine, University Hospitals Cleveland Medical Center, Cleveland, OH 44106;; ^j^Center for the Prevention and Treatment of Visual Loss, Veterans Affairs Medical Center, Iowa City, IA 52246;; ^k^Department of Ophthalmology and Visual Sciences, The University of Iowa, Iowa City, IA 52242;; ^l^Weill Cornell Autism Research Program, Weill Cornell Medicine of Cornell University, New York, NY 10065;; ^m^Department of Neuroscience, School of Medicine, Case Western Reserve University, Cleveland, OH 44106

**Keywords:** blood–brain barrier, traumatic brain injury, inflammation, neurodegeneration, neuroprotection

## Abstract

Chronic neurodegeneration in survivors of traumatic brain injury (TBI) is a major cause of morbidity, with no effective therapies to mitigate this progressive and debilitating form of nerve cell death. Here, we report that pharmacologic restoration of the blood–brain barrier (BBB), 12 mo after murine TBI, is associated with arrested axonal neurodegeneration and cognitive recovery, benefits that persisted for months after treatment cessation. Recovery was achieved by 30 d of once-daily administration of P7C3-A20, a compound that stabilizes cellular energy levels. Four months after P7C3-A20, electron microscopy revealed full repair of TBI-induced breaks in cortical and hippocampal BBB endothelium. Immunohistochemical staining identified additional benefits of P7C3-A20, including restoration of normal BBB endothelium length, increased brain capillary pericyte density, increased expression of BBB tight junction proteins, reduced brain infiltration of immunoglobulin, and attenuated neuroinflammation. These changes were accompanied by cessation of TBI-induced chronic axonal degeneration. Specificity for P7C3-A20 action on the endothelium was confirmed by protection of cultured human brain microvascular endothelial cells from hydrogen peroxide-induced cell death, as well as preservation of BBB integrity in mice after exposure to toxic levels of lipopolysaccharide. P7C3-A20 also protected mice from BBB degradation after acute TBI. Collectively, our results provide insights into the pathophysiologic mechanisms behind chronic neurodegeneration after TBI, along with a putative treatment strategy. Because TBI increases the risks of other forms of neurodegeneration involving BBB deterioration (e.g., Alzheimer’s disease, Parkinson’s disease, vascular dementia, chronic traumatic encephalopathy), P7C3-A20 may have widespread clinical utility in the setting of neurodegenerative conditions.

## Significance

Chronic neurodegeneration, a major cause of the long-term disabilities that afflict survivors of traumatic brain injury (TBI), is linked to an increased risk for late-life neurodegenerative disorders, including Alzheimer’s disease, Parkinson’s disease, vascular dementia, and chronic traumatic encephalopathy. Here, we report on the restoration of blood–brain barrier (BBB) structure and function by P7C3-A20 when administered 12 mo after TBI. This pharmacotherapy was associated with cessation of chronic neurodegeneration and recovery of normal cognitive function, benefits that persisted long after treatment cessation. Pharmacologic renewal of BBB integrity may thus provide a new treatment option for patients who have suffered a remote TBI, or other neurological conditions associated with BBB deterioration.

Traumatic brain injury (TBI) is commonly caused by motor vehicle accidents, falls, contact sports, explosions, or assaults, with an estimated 70 million people worldwide sustaining a TBI every year (1). In the United States alone, there are almost 3 million annual emergency department visits for TBI treatment (2) and ∼5 million people living with TBI-related disabilities, translating to an annual cost of ∼$80 billion (3–5). Many TBI survivors experience chronic diffuse axonal degradation and nerve cell death (6–8) associated with sensorimotor impairment, cognitive dysfunction, and emotional dysregulation, as well as increased risk of developing Alzheimer’s disease (AD), Parkinson’s disease (PD), vascular dementia, and chronic traumatic encephalopathy (CTE) (9, 10).

The initial clinical management of TBI is confined to acute measures, such as reducing intracranial pressure and edema (11), while maintaining oxygenation and meeting metabolic demand of the injured brain (12, 13). For survivors, the only chronic treatment options are prolonged physical and cognitive rehabilitation accompanied by symptom-driven medication. Unfortunately, such approaches rarely slow the long-term deterioration in neurologic function (14). In essence, TBI produces a chronic pathology that is triggered by injury-initiated persistent neurodegeneration and leads to life-long detrimental effects on multiple health outcomes (6, 15).

**Fig. 1.**
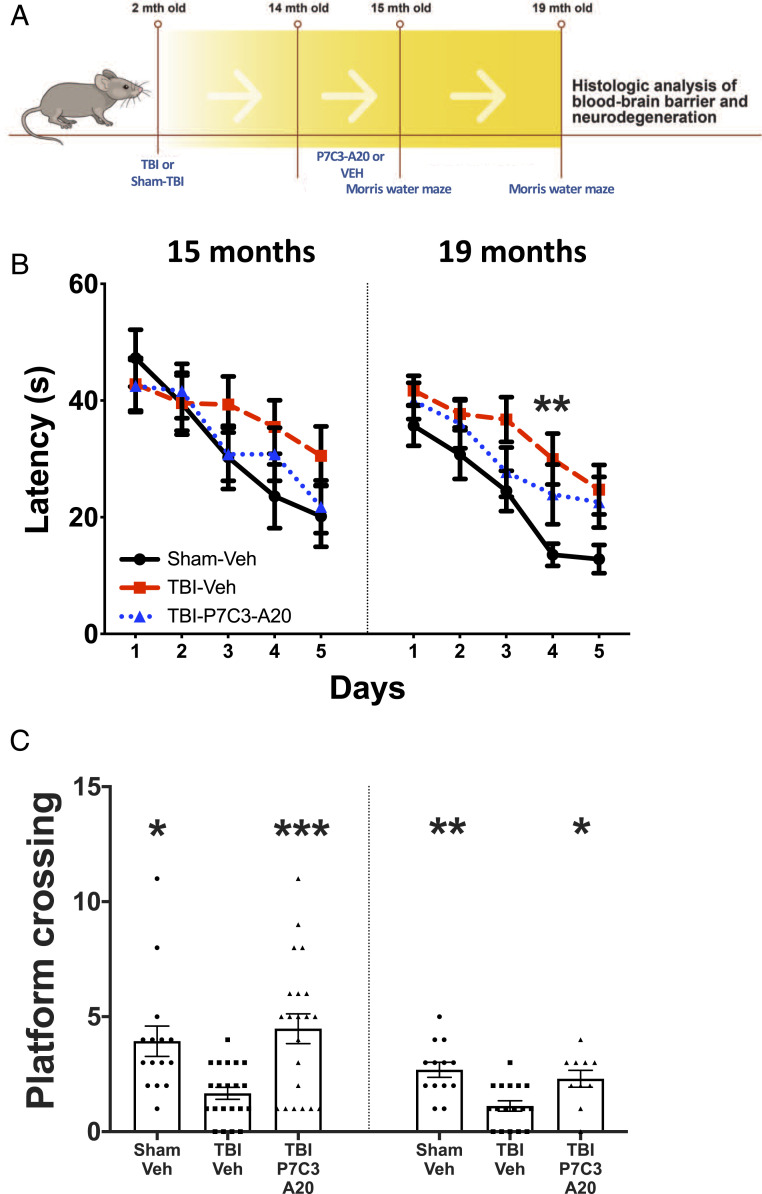
P7C3-A20 restores cognition in chronic TBI. (*A*) Experimental schematic. (*B*) All groups showed equal learning at 15 mo. At 19 mo, TBI-Veh exhibited learning deficits on day 4 compared to Sham-Veh. (*C*) Platform crossings in the memory probe test show expected aging-related decline in Sham-Veh mice, and TBI-Veh showed significant impairment relative to Sham-Veh. Memory function was fully restored in TBI-P7C3-A20 at both time points. Values are mean ± SEM. Individual data points represent individual animals. Significance was determined by repeated-measure two-way ANOVA for learning, one-way ANOVA for memory, and Dunnett’s post hoc analysis. *P < 0.05, **P < 0.01, ***P < 0.001, relative to TBI-Veh.

Despite decades of research, there remains a tremendous unmet need for new disease-modifying therapies that can mitigate post-TBI chronic neurodegeneration (16). Recent work elegantly summarized by Sandsmark et al. (17) has identified injury to the neurovascular unit (NVU) as a potential driving force for chronic neurodegeneration after TBI. Damage to the endothelial lining of the brain microvessels is observed in both human patients and mouse TBI models (18), resulting in persistent deterioration of the blood–brain barrier (BBB) and chronic brain inflammation (19, 20). Here, we have studied this chronic condition in a murine model of multimodal TBI (mmTBI) that entails jet-flow exposure in an overpressure chamber to produce globally compressive forces along with a variable amount of acceleration–deceleration and early blast wave exposure. This laboratory model produces neurodegeneration and neurobehavioral deficits reminiscent of TBI in people (21–24). We treated and analyzed mice beginning 1 y after a single injury, as outlined in Fig. 1*A*. This late time point was selected because it represents approximately the midpoint of a typical mouse’s lifespan and thus can be considered a model of delaying initiation of treatment in people until decades after their TBI. This is important because of the great many people living today who are suffering from the chronic deficits of TBI.

## Results

Two-month-old mice were subjected to TBI or sham-TBI and housed under standard conditions for 1 y. Mice were then administered either vehicle (TBI-Veh, Sham-Veh) or P7C3-A20 (10 mg⋅kg^−1^⋅day^−1^ intraperitoneally [IP]; TBI-P7C3-A20) for 4 wk. P7C3-A20 is an aminopropyl carbazole that elevates cellular nicotinamide adenine dinucleotide, enhances survival of adult-born young hippocampal neurons, and has been shown to preserve mature neurons and improve cognition in various models of nervous system disease and injury (25–45). Immediately after treatment completion, cognitive function in the 15-mo-old animals was evaluated through Morris water maze (MWM) testing. Initial task learning was equivalent between groups (Fig. 1*B*). However, significant memory deficits were recorded in the TBI-Veh group while memory performance in the TBI-P7C3-A20 mice was equivalent to the control animals (Sham-Veh) (Fig. 1*C*).

**Fig. 2.**
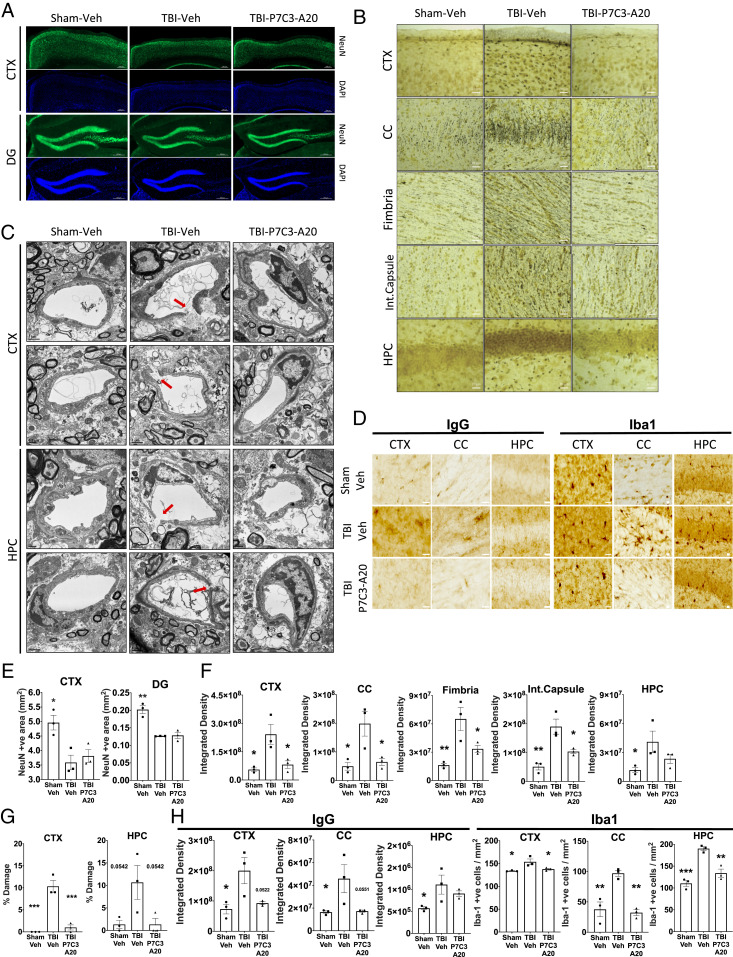
P7C3-A20 restores the BBB and arrests neurodegeneration in chronic TBI. (*A*) Neuronal cell loss measured by immunostaining for NeuN in cortex (CTX) and hippocampal dentate gyrus (DG) reveals reduced neurons in TBI-Veh and TBI-P7C3-A20 relative to Sham-Veh. (Scale bars: 200 μm.) (*B*) Ongoing neurodegeneration measured by silver staining of CTX, corpus callosum (CC), fimbria, internal capsule, and hippocampus (HPC) demonstrate significant increase in TBI-Veh relative to Sham-Veh, with TBI-P7C3-A20 restored to Sham-Veh levels. (Scale bars: 20 μm.) (*C*) Transmission electron microscopy of CTX and HPC shows BBB capillary endothelium breaks (red arrows) in TBI-Veh, but not in Sham-Veh or TBI-P7C3-A20. (Scale bars: 0.5 μm and 1 μm). (*D*) Peripheral IgG infiltration into the brain, and neuroinflammation via Iba1 microglial activation, are increased in CTX, CC, and HPC in TBI-Veh compared to Sham-Veh, and restored to Sham-Veh levels in TBI-P7C3-A20. (Scale bars: 20 μm.) (*E*) Quantification of NeuN. (*F*) Quantification of silver staining. (*G*) Quantification of BBB capillary endothelium breaks was determined by number of capillary endothelial cell (EC) breaks per an average of 113 capillaries. (*H*) Quantification of IgG infiltration and Iba1 microglial activation. All values are mean ± SEM. Individual data points represent individual animals. Significance was determined by one-way ANOVA and Dunnett’s post hoc analysis. *P < 0.05, **P < 0.01, ***P < 0.001 relative to TBI-Veh.

**Fig. 3.**
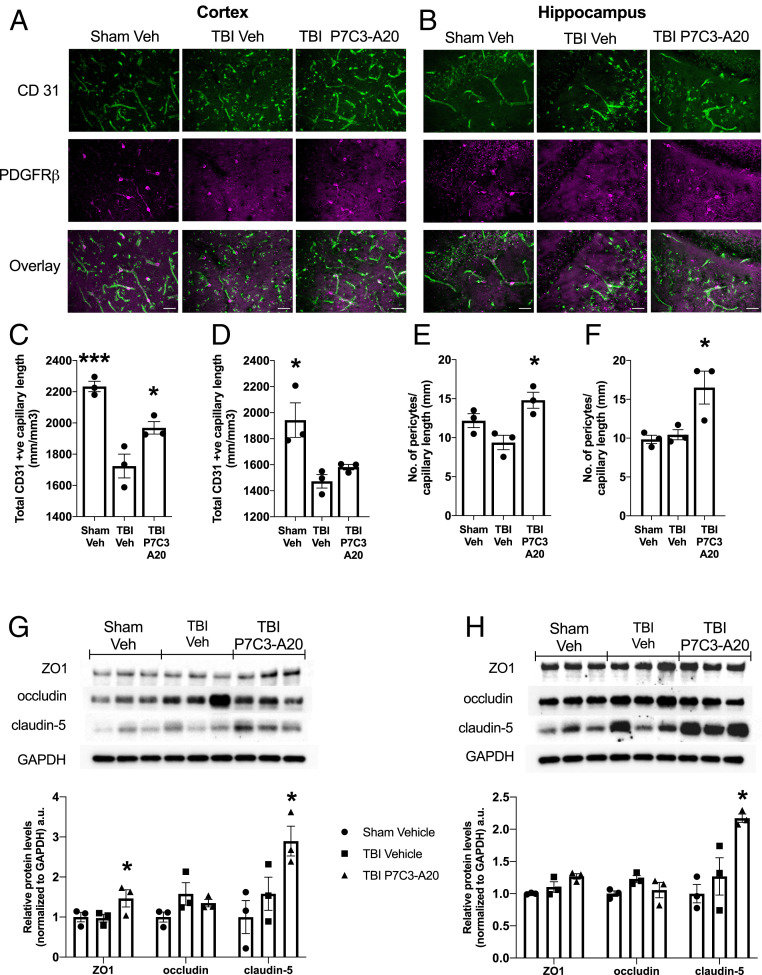
P7C3-A20 restores BBB capillary endothelium length and tight junction protein expression in chronic TBI and also increases basal pericyte abundance. (*A–F*) CD31 staining was used to determine capillary endothelium length, and PDGFRβ staining was used to quantify pericytes. Representative pictures (*A* and *B*) and quantification (*C–F*) from CTX and HPC of Sham-Veh, TBI-Veh, and TBI-P7C3-A20 show reduced capillary endothelium length in TBI-Veh relative to Sham-Veh, which is restored to normal by P7C3-A20. TBI-P7C3-A20 shows an increase in the number of pericytes per capillary length in the CTX and the HPC relative to both Sham-Veh and TBI-Veh. (Scale bars: 25 μm.) (*G* and *H*) Western blot analysis of tight junction proteins reveals that TBI-P7C3-A20 animals have higher levels of claudin-5 in the CTX and HPC, and of ZO1 in the CTX. Values are mean ± SEM. Individual data points represent individual animals. Significance was determined by one-way ANOVA and Dunnett’s post hoc analysis, *n* = 3; *P < 0.05, ***P < 0.001 relative to TBI-Veh. a.u., arbitrary unit.

Animals were then housed under standard conditions with no treatment for four additional months (Fig. 1*A*). At 19 mo of age (17 mo postinjury), TBI-Veh mice exhibited impaired learning (Fig. 1*B*) and memory (Fig. 1*C*) whereas TBI-P7C3-A20 mice again performed as well as Sham-Veh animals (Fig. 1 *B* and *C*). TBI-dependent motor slowing was not corrected (*SI Appendix*, Fig. S1). While similar degrees of hippocampal and cortical neuronal cell loss were observed in both TBI groups (Fig. 2 *A* and *E*), chronically active neurodegeneration (as quantified by silver staining of axonal degeneration) was arrested by P7C3-A20 treatment (Fig. 2 *B* and *F*). Assessment of the NVU by transmission electron microscopy revealed cortical and hippocampal BBB capillary endothelium breaks in TBI-Veh mice that were absent in Sham-Veh– and TBI-P7C3-A20–treated animals (Fig. 2 *C* and *G* and *SI Appendix*, Figs. S2 and S3). Immunohistochemical examination conducted in parallel showed that the prior P7C3-A20 treatment had also reduced infiltration of peripheral immunoglobulin G (IgG) (a marker of BBB degradation) and decreased the number of Iba1^+^ cells (a marker of activated microglia in neuroinflammation) (Fig. 2 *D* and *H*). P7C3-A20 also restored BBB endothelium length toward that seen in Sham-Veh brains (Fig. 3 *A*–*D*). Further benefits of P7C3-A20 on the BBB included increases in capillary pericyte density (Fig. 3 *A*, *B*, *E*, and *F*) and increases in the expression of BBB tight junction protein claudin-5 in the cortex and hippocampus, and of zona occludens-1 in the cortex (Fig. 3 *G* and *H*).

**Fig. 4.**
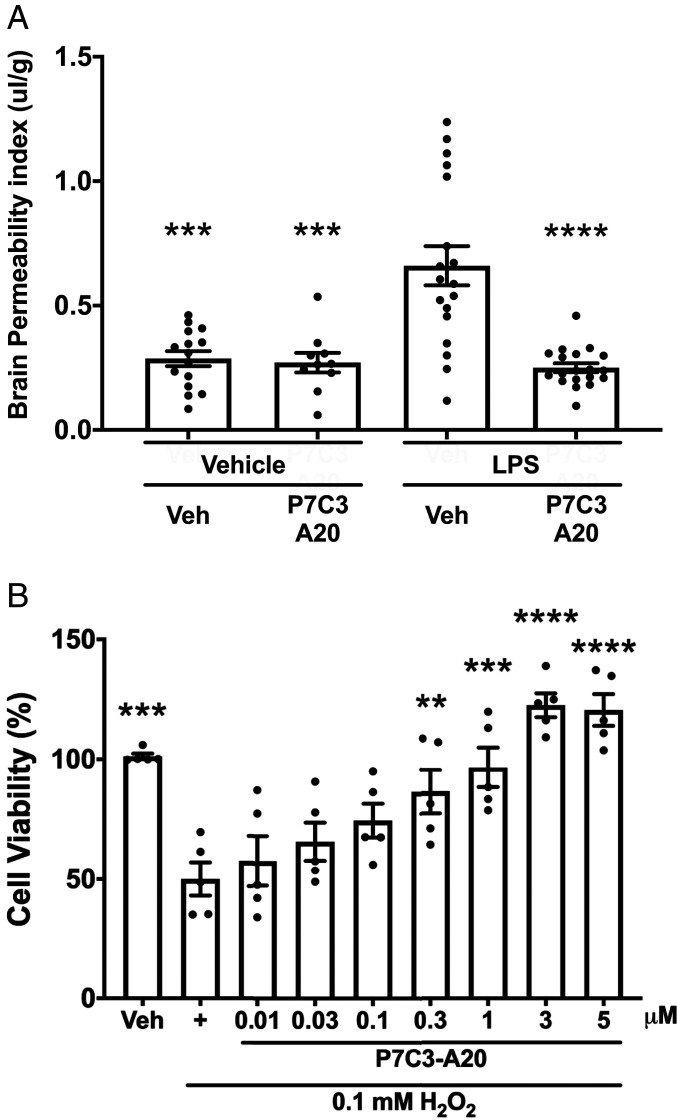
P7C3-A20 protects endothelial cells in vivo and in vitro. (*A*) LPS-damage to the BBB was measured by brain permeability of fluorescent-conjugated 3-kDa dextran, with greater dextran entry in LPS-Veh relative to Veh-Veh. LPS-P7C3-A20, however, showed significant reduction relative to LPS-VEH. Values are mean ± SEM. Individual data points represent individual animals. Significance was determined by one-way ANOVA and Tukey’s post hoc analysis. ***P < 0.001, ****P < 0.0001 relative to Veh-LPS. (*B*) Cultured human brain microvascular endothelial cells exposed to 0.1 mM H_2_O_2_ showed 50% reduction in cell viability, which was blocked by P7C3-A20 in a dose–response manner. Values are presented as mean ± SEM. Individual data points represent individual experiments of six replicates. Significance was determined by one-way ANOVA and Dunnett’s post hoc analysis, *n* = 5; **P < 0.01, ***P < 0.001, ****P < 0.0001 relative to H_2_O_2_ cells treated only.

To confirm that P7C3-A20 was directly protecting brain endothelial cells, we dosed separate cohorts of mice with lipopolysaccharide (LPS), which damages the BBB (46). Twelve hours after inoculation, the treatment group received a single dose of P7C3-A20 (10 mg/kg IP). BBB integrity was assessed in both groups 12 h later by quantifying central nervous system (CNS) permeation of fluorescent-conjugated 3-kDa dextran (47). Notably, LPS-mediated entry of dextran was blocked in the P7C3-A20–treated mice (Fig. 4*A*). P7C3-A20 also directly protected cultured human microvascular endothelial cells from hydrogen peroxide-mediated cell death (Fig. 4*B*), providing further evidence of this aminopropyl carbazole’s ability to preserve endothelial health and function.

**Fig. 5.**
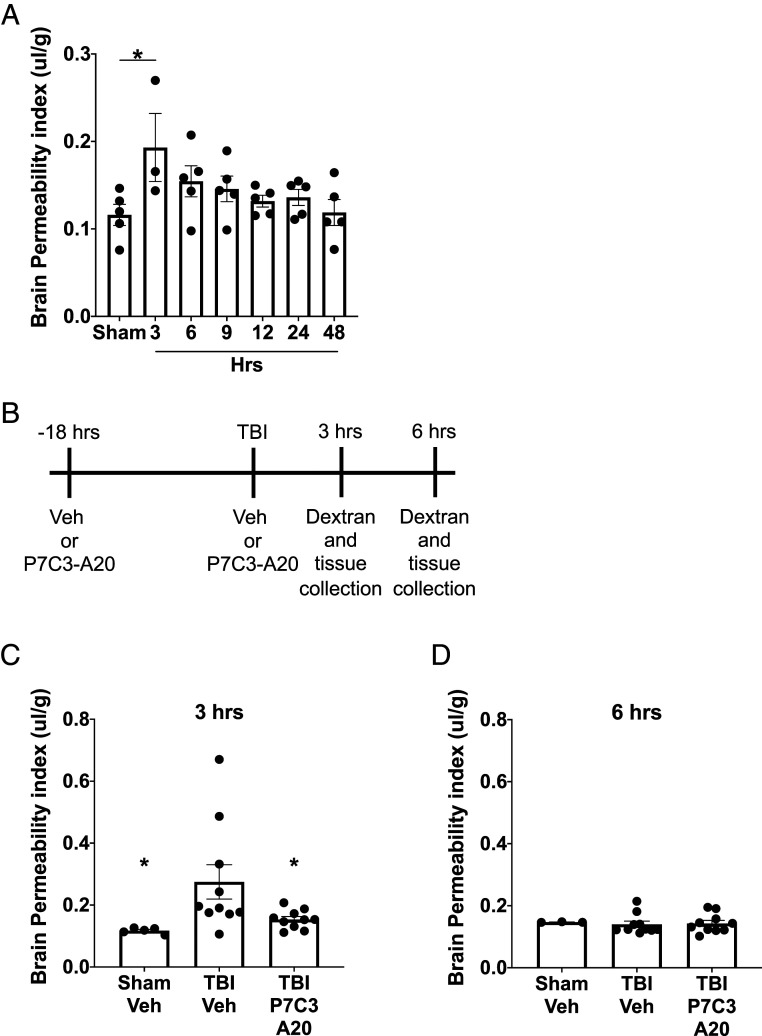
P7C3-A20 treatment acutely protects BBB integrity after TBI. (*A*) Acute damage to the BBB was determined by brain permeability of fluorescent-conjugated 3-kDa dextran at different time points after TBI, with significantly higher dextran permeability at 3 h after the injury compared against Sham group, and returning to normal thereafter. Values are mean ± SEM. Individual data points represent individual animals. Significance was determined by one-way ANOVA and Dunnett’s post hoc analysis, *P < 0.05 relative to Sham. (*B*) Experimental schematic. Efficacy of P7C3-A20 in protecting the BBB was determined at (*C*) 3 h and (*D*) 6 h after TBI. Values are mean ± SEM. Individual data points represent individual animals. Significance was determined by one-way ANOVA and Dunnett’s post hoc analysis, *P < 0.05 relative to TBI-Veh.

Lastly, we wondered whether treatment with P7C3-A20 would protect the BBB in acute TBI as well. Both we and others (39, 48–53) have previously observed in different models of TBI that acute disruption of the BBB returns to normal at variable times after injury. Thus, we first established the pattern of acute BBB degradation in our model of mmTBI. As shown in Fig. 5*A**,* CNS permeation of fluorescent-conjugated 3-kDa dextran approximately doubled 3 h after TBI and returned to sham levels at subsequent time points of 6, 9, 12, 24, and 48 h. We then treated mice with either Vehicle or P7C3-A20 (10 mg/kg IP) 18 h before TBI and again at the time of TBI (Fig. 5*B*). Here, TBI-Veh mice showed approximately doubled BBB permeability compared to Sham-Veh mice at the 3-h time point, as expected from the previous time course experiment, and TBI-P7C3-A20 mice were fully protected from this BBB degradation (Fig. 5*C*). At the 6-h time point, none of the groups showed any impairment in BBB function (Fig. 5*D*), as expected.

## Discussion

Proper CNS function is critically dependent on an extensive vascular network that works in concert with multiple cell types to form the NVU. The NVU rapidly responds to the changing metabolic needs of the brain, while also protecting the brain parenchyma from exposure to injurious agents via the BBB. The BBB prevents entry of peripheral toxins, while also mediating the removal of proteins and other substances from the brain parenchyma. Through continuous crosstalk, the NVU forms an integrated system of neurovascular coupling that ensures optimal supply of oxygen and micronutrients across the BBB consonant with the metabolic demand that varies with neuronal activity. Disruption of the NVU and the BBB that it maintains can harm brain health, potentially leading to neurodegeneration (54, 55). Here, we show that deterioration of brain microvascular endothelial cells accompanies chronic axonal degeneration 1 y after TBI in mice. This is consistent with human studies of chronic TBI. For example, in one study, 47% of brains from long-term TBI survivors (up to 47 y after injury) showed histologic evidence of BBB deterioration (56). Furthermore, decreased expression of claudin-1 and zona occludens-1 tight junction proteins and immunoglobulin extravasation into brain parenchyma were observed in brain tissue from a patient with chronic traumatic encephalopathy (57), and multiple imaging modalities have demonstrated chronically compromised cerebral blood flow dynamics in patients with chronic TBI (17).

We also show here that P7C3-A20 directly protects brain microvascular endothelial cells in vivo in mice and in cultured human cells. Taken together, our results suggest that BBB deterioration may be a major contributor to chronic neurodegeneration after remote TBI and that its repair may halt this pathology. Although the mechanism and timing of damage to the BBB in our model remain to be further studied, our results provide a rational foundation for developing treatments for patients suffering from chronic progressive neurodegeneration and cognitive dysfunction after remote TBI. This is critical for the field as, despite the enormous societal burden of chronic neurodegeneration after TBI, there are currently no medicines that prevent or slow this disease process. We note that it is currently not possible discern the potentially complementary beneficial effects of P7C3-A20 on both endothelial cells and neurons. Future pharmacologic tools that act selectively on endothelial cells or neurons might be able to resolve this question. At present, our results support the broad potential utility of a pharmacologic agent able to directly repair the BBB, a property that we demonstrate here is possessed by P7C3-A20.

Lastly, it is important to place into context how these results relate to the association between TBI and later neurodegenerative disease. Patients with AD and AD-related dementias frequently display increased BBB permeability (58), and TBI is a well-recognized major risk factor for AD and other dementias, including Parkinson’s disease, vascular dementia, and chronic traumatic encephalopathy (10, 54, 59–63). BBB deterioration has also been observed in these same neurodegenerative conditions (10, 64, 65). Recently, a blood biomarker of BBB deterioration was also proposed for AD (66), and the two-hit vascular hypothesis of AD proposes the BBB disruption is an initiating event in amyloid beta deposition in the brain (54). In support of this, human studies have identified alteration in BBB permeability at the earliest stages of cognitive decline and AD (67, 68). How the trajectory of BBB dysfunction after TBI proceeds from acute impairment and recovery, to later chronic deterioration, is currently unknown and is an important question for the field. The astrocyte-derived lipid transport molecule apolipoprotein-e (apoE) is a major factor that regulates BBB permeability, via regulation of low density lipoprotein receptor-related protein 1-cyclophilin A-matrix metalloproteinase-9 signaling in pericytes at the NVU (69). It is interesting to note that the apoE4 allele, one of the strongest known genetic risk factors for AD (70), also impairs spontaneous BBB repair after TBI (48) and has been demonstrated as epidemiologically synergistic with TBI for increased risk of AD (71). In conclusion, we speculate that restoration of BBB integrity with P7C3-A20 after TBI could yield not only cognitive benefits but also reduce the otherwise increased risk for patients to develop other forms of neurodegenerative disease later in life.

## Materials and Methods

### Animals.

Seven-week-old, male, C57BL/6J (stock no. 000664) mice were obtained from The Jackson Laboratory. All mice were maintained group-housed with water and food provided ad libitum under humidity, light (12-h light/dark cycle), and temperature-controlled conditions. All animal procedures were performed in accordance with the protocol approved by the University of Iowa Institutional Animal Care and Use Committee and the Louis Stokes Cleveland Veterans Affairs (VA) Medical Center Institutional Animal Care and Use Committee.

### Drug Administration.

P7C3-A20 was dissolved in 2.5% vol of dimethyl sulfoxide (DMSO), followed by addition of 10% vol Kolliphor (Sigma-Aldrich) and vigorous vortexing. The solution was then diluted in 87.5% vol of D5W (filtered solution of 5% dextrose in water, pH 7.0). P7C3-A20 was administered IP daily for 1 mo, beginning 1 y after TBI.

### mmTBI Model.

Eight-week-old male C57BL/6J mice were anesthetized with ketamine (100 mg/kg) and xylazine (10 mg/kg) via IP injection and placed in an enclosed chamber constructed from an air tank partitioned into two sides and separated by a port covered by a mylar membrane. The pressure in the side not containing the mouse was increased to cause membrane rupture at 20 pounds per square inch (PSI), which generates an ∼1.0- to 1.5-ms air jet flow of 137.9 ± 2.09 kPa that passes through the animal’s head. The head was untethered in a padded holder while the body was fully shielded by a metal tube. The jet of air produced upon membrane rupture in this apparatus produces a collimated high-speed jet flow with extreme dynamic pressure that delivers a severe compressive impulse. Variable rupture dynamics of the diaphragm through which the jet flow is originated also generate a weak and infrequent shock front. There is also a minor component of acceleration–deceleration injury to the unrestrained head as it is agitated in the headrest. The sham group was anesthetized and passed the same process except for the injury. The chronic study was begun with allocation of 15 animals to the Sham-Veh group, 25 animals to the TBI-Veh group, and 25 animals to the TBI-P7C3-A20 group. More animals were allocated to the TBI group in order to ensure survival of at least 15 per group by the end of the study. At the 15-mo time point, relative to the day of injury, the Sham-Veh group had no mortality, and the TBI-Veh and TBI-P7C3-A20 groups both showed 16% mortality. At the 19-mo time point, relative to the day of injury, the Sham-Veh group had no mortality, the TBI-Veh group showed 32% mortality, and theTBI-P7C3-A20 group showed 40% mortality.

### MWM.

MWM was conducted in a 128-cm diameter tank filled with 19 cm of room temperature water (22 °C). White nontoxic paint was added to the water to reduce platform visibility. Four different and equally spaced visual cues with different shapes and colors were placed inside the tank to use as reference in the location of a submerged 9-cm-diameter platform. Each animal passed through a training session of four trials per day (60 s per trial or until the mouse found the platform) for five consecutive days. Mice that failed to find the platform were guided to find it and allowed to stay on the platform for 30 s. During probe test on day 6, 24 h after the last training day, the platform was removed, and each animal was monitored for 60 s. Any-maze video tracking software (Stoelting Co.) was used to measure latency to find the hidden platform through the training session, platform crossing, distance traveled, and mean speed during the probe day.

### Brain Tissue Collection.

Mice were anesthetized with ketamine (100 mg/kg) and xylazine (10 mg/kg) via IP injection and transcardially perfused with phosphate-buffered saline (PBS) followed by 4% paraformaldehyde in PBS at pH 7.4. Brains were collected and postfixed in 4% paraformaldehyde in PBS at pH 7.4 overnight at 4 °C and then transferred to 30% sucrose for 72 h. Brains were cut coronally (40-μm sections), preserved in cryoprotectant, and stored at −20° before staining.

### Electron Microscopy Analysis of BBB Integrity.

Forty-micrometer-thick brain sections were washed three times with 1× PBS. Tissue was then fixed by immersion in quarter strength Karnovsky’s fixative solution for 2 h at room temperature. After washing, the specimen was postfixed for 2 h in an unbuffered 1:1 mixture of 2% osmium tetroxide and 3% potassium ferrocyanide (72). After rinsing with distilled water, specimens were soaked overnight in an acidified solution of 0.25% uranyl acetate. After another rinse in distilled water, specimens were dehydrated in ascending concentrations of ethanol, passed through propylene oxide, and embedded in an EMbed 812 embedding media (Electron Microscopy Sciences, Hatfield, PA). Thin sections (70 nm) were cut on an RMC MT6000-XL ultramicrotome. These were mounted on Gilder square 300 mesh nickel grids (Electron Microscopy Sciences) and then sequentially stained with acidified methanolic uranyl acetate, followed by a modification of Sato’s triple lead stain (72). These were coated on a Denton DV-401 carbon coater (Denton Vacuum LLC) and were examined in an FEI Tecnai Spirit (T12) with a Gatan US4000 4k × 4k resolution charge-coupled device (CCD).

### Immunohistochemistry.

BBB permeability was assessed by visualization of IgG in the brain. Free-floating sections (40 μm thick) were washed in PBS. Next, endogenous peroxidase activity and nonspecific staining were blocked (5% bovine serum albumin [BSA] plus 5% horse serum). Sections were then incubated in biotinylated anti-mouse IgG antibody (1:500, BA-2000; Vector Laboratories) overnight at 4 °C. After washing with PBS, sections were incubated with avidin–biotin complex (ABC kit, PK-4000; Vector Laboratories) and developed using 3,3′-diaminobenzidine (DAB) (DAB Peroxidase Substrate Kit, SK-4100; Vector Laboratories). All sections were incubated with the same batch of DAB, and all reactions were performed at the same time and exposed for the same amount of time. Brain sections were mounted on glass slides and coverslipped with Permount. Iba1, NeuN, CD 31, and PDGFRβ staining was performed in free-floating brain sections washed in PBS and permeabilized with 0.1% Triton X-100 or 0.25% Triton X-100 (for CD 31 and PDGFRβ) in PBS. For PDGFRβ staining, antigen retrieval was performed using Dako Agilent Target Retrieval solution (S1700 RTU). Nonspecific staining was blocked in 5% BSA and 5% horse serum (for Iba1 staining, endogenous peroxidase was also blocked). Brain sections were incubated in primary antibodies overnight at 4 °C. After washing, sections were incubated in secondary antibodies for 1 h at room temperature. Brain tissues were mounted on glass slides with VECTASHIELD antifade mounting medium (H-1000, H-1200; Vector Laboratories). The primary antibodies and dilutions used were as follow: anti-Iba1 (1:500, 019-19741; Fujifilm Wako); anti-NeuN (1:500, MABN-140; EMD Millipore Corp.); anti-cd 31 (1:100, 550274; BD Biosciences); and anti-PDGFRβ (1: 100, AF1042; R&D Systems). The secondary antibodies and dilutions used were as follows: biotinylated anti-mouse IgG (1:1,000, BA-2000; Vector Laboratories); biotinylated anti-rabbit IgG (1:1,000, BA-1000; Vector Laboratories); anti-mouse Alexa 488 (1:1,000, A32723; Invitrogen); anti-rabbit Alexa 488 (1:1,000, A21206; Invitrogen); and anti-goat Alexa 647(1:1,000, A21447; Invitrogen). Free-floating brain sections were processed and stained with an FD NeuroSilver Kit (FD NeuroTechnologies, Columbia, MD).

### Image Acquisition and Analysis.

Images were acquired using the Zeiss Axio Imager.M2 microscope and Zeiss Axio Scan.Z1, keeping the light intensity and exposure time constant. ImageJ version 1.42 software (NIH, Bethesda, MD) was used to analyze brightfield and fluorescent images. Silver staining (black staining) was quantified using the plugin of the color deconvolution method described by Ruifrok and Johnston (73). CD31 positive capillary length and pericytes coverage of these CD31 positive capillaries were imaged and analyzed following the method described in Bell et.al. (74).

### Immunoblotting.

Cortical and hippocampal tissue was dissected from flash-frozen tissue and ground in radioimmunoprecipitation assay (RIPA) buffer (R0278; Sigma-Aldrich) containing protease and phosphatase inhibitor mixture (1861284; Thermo Scientific). Lysates were centrifuged at 18,000 × *g* at 4 °C for 30 min. The protein concentration of the supernatant was measured by the bicinchoninic acid (BCA) protein assay kit (A53225; Thermo Scientific). Proteins were heated for 5 min in Laemmli sample buffer (1610737; Bio-Rad Laboratories) containing beta-mercaptoethanol. Proteins were resolved in 4 to 20% Criterion TGX Stain-free gels (5678095; Bio-Rad Laboratories) and transferred onto a 0.2-μm polyvinylidene fluoride membrane (1704157; Bio-Rad Laboratories) using the Trans-Blot Turbo system (Bio-Rad Laboratories). After transfer, membranes were blocked in 5% nonfat dry milk in Tris-buffered saline containing Tween 20 (TBST) for 1 h at room temperature. Then, membranes were incubated in primary antibodies overnight at 4 °C. The primary antibodies and dilutions used were as follow: anti-ZO1 (1:1,000, 33-9100; Thermo-Fischer); anti-occludin (1:500, B611091; BD Biosciences); anti–claudin-5 (1:1,000, 352500; Thermo-Fischer); and anti-GAPDH (1:5,000, MAB 374; EMD Millipore Corp.). After washing in TBST, membranes were incubated with horseradish peroxidase-conjugated secondary antibodies for 1 h and developed using SuperSignal West Femto Maximum Sensitivity substrate (34096; Thermo Scientific). ImageJ version 1.42 software (NIH, Bethesda, MD) was used to densitometry analysis.

### LPS-Induced BBB Damage Assay.

BBB damage was induced by IP injection of 3 mg/kg LPS from *Escherichia coli* (O111:B4, LPS25; Sigma) dissolved in saline. The damage was assessed 24 h after LPS injection using a dextran extravasation assay. The dose and duration of this assay were selected based on Banks et al. (46). For the dextran extravasation assay, we used 3-kDa TMR-dextran (D3308; Invitrogen) and followed the method described in Devraj et al. (47) with one modification—dextran was injected retroorbitally, and therefore blood/brain was collected 5 min after dextran injection. P7C3-A20 (10 mg/kg, IP) was given 12 h after LPS injection.

### Cytotoxicity Assay on Human Brain Microvascular Endothelial Cells.

Primary human brain microvascular cells were obtained from Cell Systems Corporation (cat. no. ACBRI 376) and maintained according to the manufacturer’s guidelines. Briefly, cells were cultured in Complete Classic Medium (cat. no. 4Z0-500). Cells were incubated at 37 °C in 95% air and 5% CO_2_ in a humidified incubator. Cells were grown up to 80 to 85% confluence and then seeded into a 96-well plate (1 × 10^5^ cells per well) for cell viability analysis. Cells of passage number 7 to 11 were seeded into a 96-well plate with complete serum media and grown for 24 h before treatment. Immediately prior to treatment, medium with serum was removed, and Complete Serum-Free Medium (cat no. SF-4Z0-500; Cell Systems) with 2% fetal bovine serum (FBS) was added to each well. P7C3-A20 was dissolved in DMSO, and further dilutions were made in the serum-free medium with 2% FBS. Cells were treated with 0.1 and 0.2 mM H_2_O_2_, and different concentrations of P7C3-A20 (i.e., 0.03, 0.1, 0.3, 1 and 5 μM). Control and H_2_O_2_-treated cells were also matched with vehicle concentration: i.e., 0.05% DMSO. Cells were incubated for 24 h after treatment, and cytotoxicity was measured by a CyQUANT Direct Cell Proliferation Assay Kit (cat. no. C35011), as per the manufacturer’s protocol. A standard curve was prepared during each experiment with a known amount of cells, and relative cytotoxiciy in each treatment group was calculated as the percentage of control cells (i.e., relative fluorescence unit in control cells with 2% FBS was considered as 100%).

### Statistical Analysis.

Values are presented as mean ± SEM. Statistical analyses were done using GraphPad Prism, Version 8.3.0.

## Supplementary Material

Supplementary File

## Data Availability

All study data are included in the article and *SI Appendix*.
