# Supplementary material for: P7C3-A20 treatment one year after TBI in mice repairs the blood–brain barrier, arrests chronic neurodegeneration, and restores cognition
Source: Proc Natl Acad Sci U S A. 2020 Oct 21;117(44):27667–75. doi: 10.1073/pnas.2010430117 (PMC7959512; doi:10.1073/pnas.2010430117)
Supplement: Supplementary File [file pnas.2010430117.sapp.pdf]

Supplemental Information for

P7C3-A20 protects the blood-brain barrier after systemic inflammation or traumatic brain injury (TBI), and treatment one year after TBI arrests chronic neurodegeneration and restores cognition

Edwin Vázquez-Rosa[1,2†], Min-Kyoo Shin[1,2†], Matasha Dhar[1,2†], Kalyani Chaubey[1,2], Coral J. Cintrón-Pérez[1,2], Xinmiao Tang[3], Xudong Liao[3], Emiko Miller[1,2], Yeojung Koh[1,2], Sarah Barker[1,2], Kathryn Franke[1,2], Danyel R. Crosby[1,2], Rachel Schroeder[4], Josie Emery[4], Terry C. Yin[4], Hisashi Fujioka[5], James D. Reynolds[1,6,7], Matthew M. Harper[8], Mukesh K. Jain[1,3], Andrew A. Pieper[1,2,6,8,9,10]

[1]Harrington Discovery Institute, University Hospitals Cleveland Medical Center, Cleveland, OH 44106 USA
[2]Department of Psychiatry Case Western Reserve University, Geriatric Research Education and Clinical Centers, Louis Stokes Cleveland VAMC, Cleveland, OH 44106 USA
[3] Department of Medicine, Case Cardiovascular Research Institute, Case Western Reserve University, 10900 Euclid Avenue, Cleveland, OH 44106, USA; Harrington Heart and Vascular Institute, University Hospitals Cleveland Medical Center, 2103 Cornell Road, Cleveland, OH 44106, USA
[4]The Department of Psychiatry, University of Iowa, Iowa City IA
[5]Cryo-EM Core, Case Western Reserve University School of Medicine, Cleveland, OH, USA
[6]Institute for Transformative Molecular Medicine, School of Medicine, Case Western Reserve University, Cleveland, OH, USA
[7]Departments of Anesthesiology & Perioperative Medicine, University Hospitals Cleveland Medical Center, Cleveland, OH, USA
[8]Veterans Affairs Medical Center, Center for the Prevention and Treatment of Visual Loss, Iowa City IA; The University of Iowa Departments of Ophthalmology and Visual Sciences, Iowa City, IA USA
[9]Weill Cornell Autism Research Program, Weill Cornell Medicine of Cornell University, NY, NY, USA
[10]Department of Neuroscience, Case Western Reserve University, School of Medicine, Cleveland, OH, USA

Address for Correspondence:
Dr. Andrew A. Pieper, MD, PhD
Harrington Discovery Institute
University Hospitals of Cleveland Medical Center
Department of Psychiatry, Case Western Reserve University
Louis Stokes VA Medical Center of Cleveland
Tel 216-368-4273
Email: Andrew.Pieper@HarringtonDiscovery.org

†These authors contributed equally: Edwin Vázquez-Rosa, Min-Kyoo Shin, Matasha Dhar

Classification
Major: Biological Sciences
Minor: Neuroscience

Keywords: Blood-brain barrier, traumatic brain injury, lipopolysaccharide, neurodegeneration, neuroprotection, endothelium, Alzheimer's disease, Parkinson's disease

Author Contributions

A.A.P., E.V-R, M-K.S, M.D., and K.C conceived the study, designed experiments, and interpreted data. E.V-R., M-K.S, M.D., K.C., C.C-P., X.T., X.L., E.M., Y.K., D.R.C., R.S., J.E., T.C.Y., S.B., K.F. conducted experiments. H.F. conducted electron microscopy experiments and analysis. A.A.P., J.D.R., M.M.H., M.K.J., E.V-R., M-K.S., and M.D. wrote the manuscript. All authors reviewed and approved the manuscript.

SI Fig. 1.

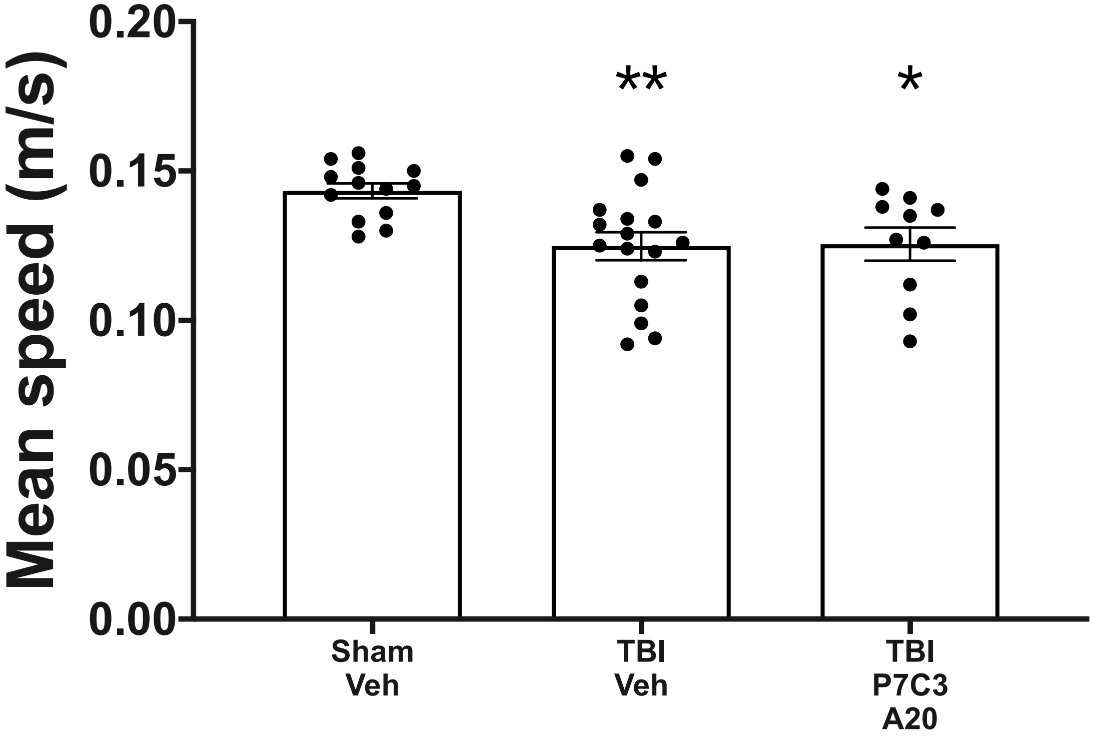

SI Fig. 1. P7C3-A20 does not restore normal swim speed in chronic TBI. Mean swimming speed was chronically reduced after TBI at 19 months, and this deficit was not ameliorated by P7C3-A20. Values are mean ± SEM. Individual data points represent individual animals. Significance was determined by one-way ANOVA and Tukey's post hoc analysis, * $p < 0.05$, ** $p < 0.01$ relative to Sham-Veh.

SI Fig. 2.

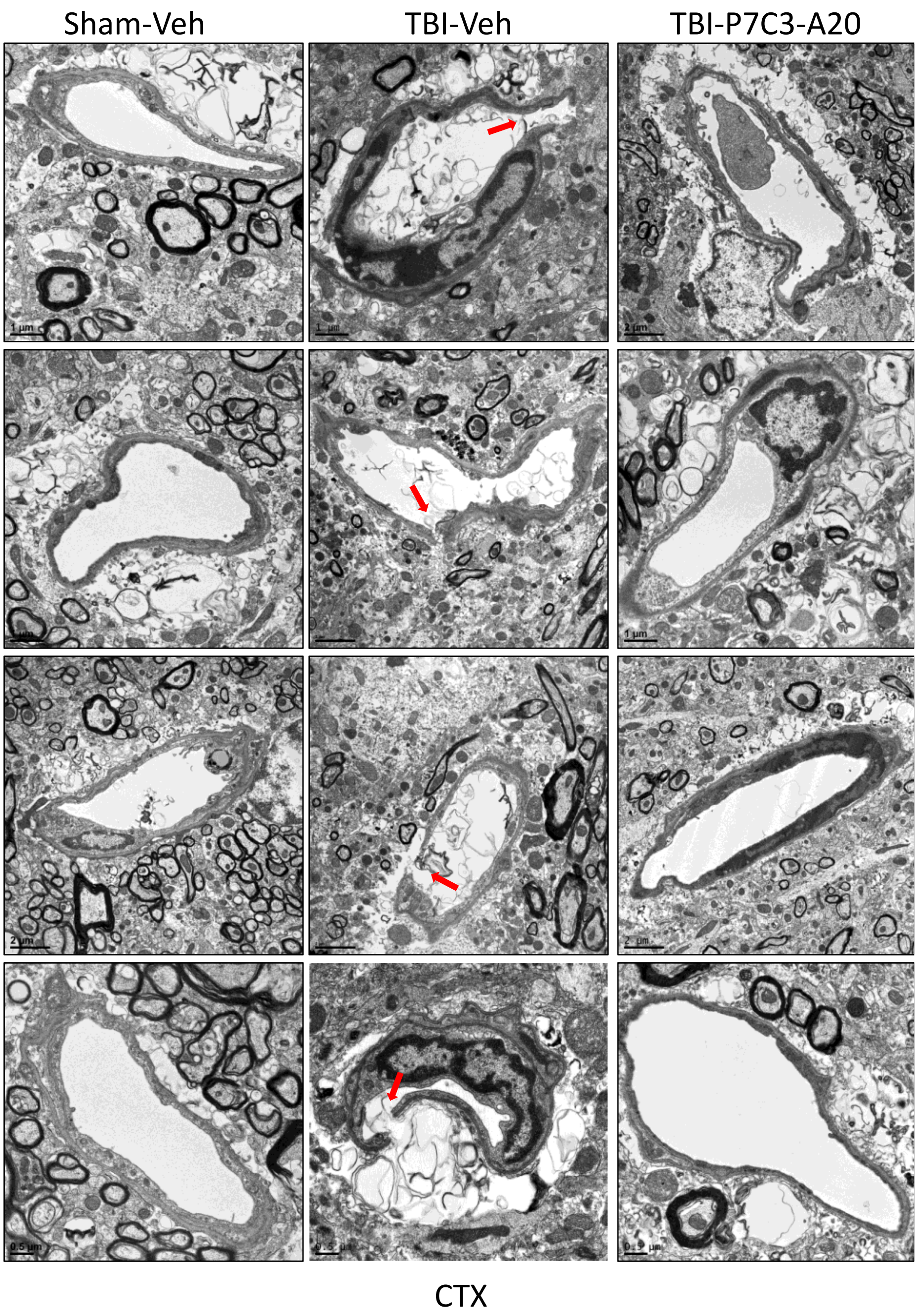

SI Fig. 2. Representative transmission electron microscopy images from the cerebral cortex of Sham-Veh, TBI-Veh and TBI-P7C3-A20 animals. Red arrows indicate breaks in endothelium.

SI Fig 3.

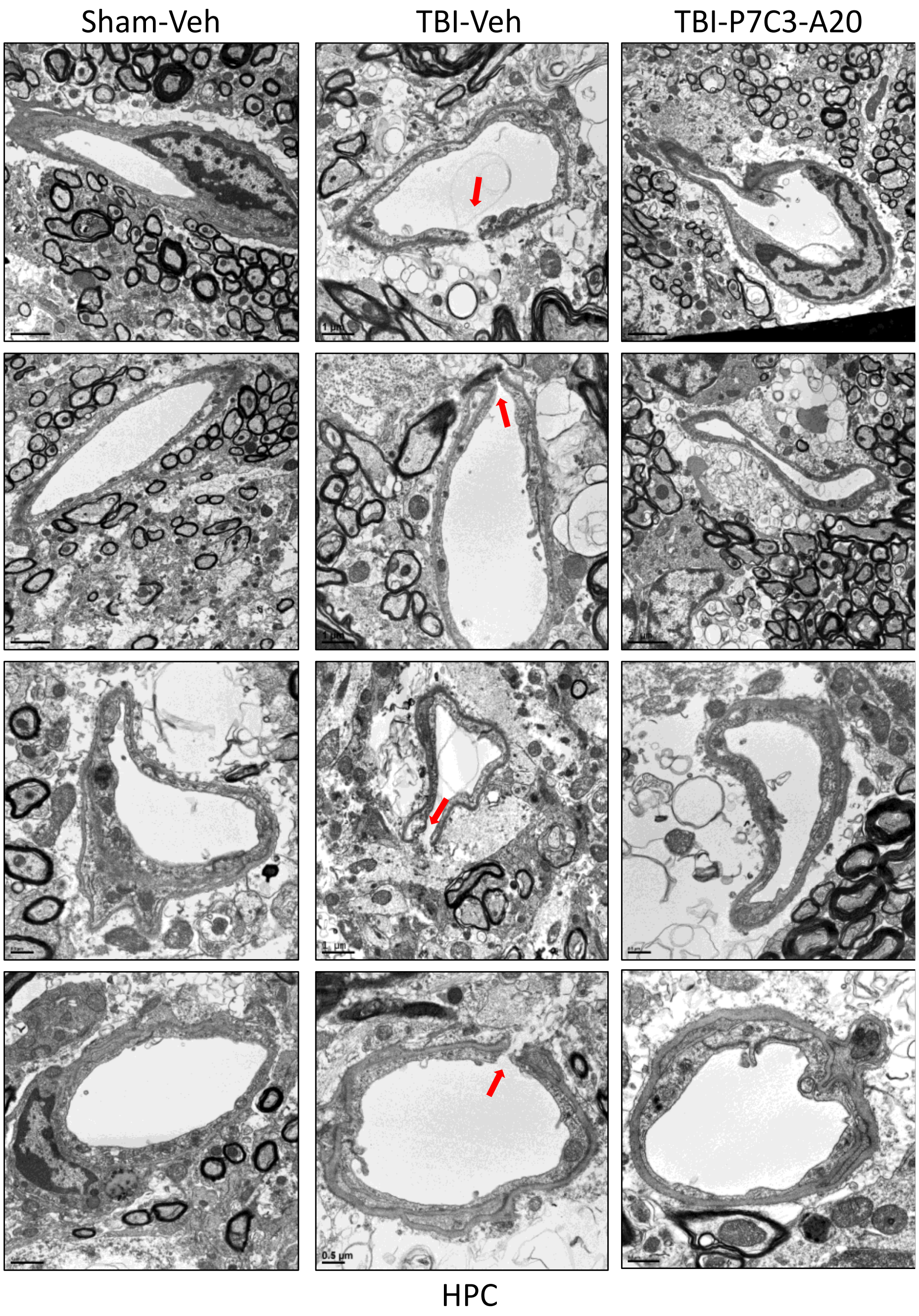

SI Fig 3. Representative transmission electron microscopy images from the hippocampus of Sham-Veh, TBI-Veh and TBI-P7C3-A20 animals. Red arrows indicate breaks in endothelium.
